# The Role of ^18^F-FDG-PET and PET/CT in Patients with Colorectal Liver Metastases Undergoing Selective Internal Radiation Therapy with Yttrium-90: A First Evidence-Based Review

**DOI:** 10.1155/2014/879469

**Published:** 2014-02-02

**Authors:** Salvatore Annunziata, Giorgio Treglia, Carmelo Caldarella, Federica Galiandro

**Affiliations:** ^1^Department of Bioimaging and Radiological Sciences, Institute of Nuclear Medicine, Catholic University of the Sacred Heart, Largo Agostino Gemelli 8, 00168 Rome, Italy; ^2^Department of Nuclear Medicine and PET Center, Oncology Institute of Southern Switzerland, via Ospedale 12, 6500 Bellinzona, Switzerland; ^3^School of Medicine, Catholic University of the Sacred Heart, Largo Agostino Gemelli 8, 00168 Rome, Italy

## Abstract

*Purpose*. To provide a first evidence-based review of the literature on the role of fluorine-18-fluorodeoxyglucose positron emission tomography and positron emission tomography/computed tomography (FDG-PET and PET/CT) in patients with colorectal liver metastases (CRLM) undergoing selective internal radiation therapy (SIRT) with yttrium-90 (^90^Y) microspheres. *Methods*. A comprehensive computer literature search was conducted to find relevant published articles on whole-body FDG-PET or PET/CT in patients with CRLM undergoing SIRT. *Results*. We identified 19 studies including 833 patients with CRLM undergoing SIRT. The role of FDG-PET or PET/CT was analysed in treatment planning, treatment response evaluation, and as prognostic tool. *Conclusion*. FDG-PET and PET/CT provide additional information in treatment evaluation of CRLM patients treated with SIRT and may have a role in treatment planning and patient selection. FDG-PET/CT is emerging as good prognostic tool in these patients.

## 1. Introduction

Liver is the most frequent site of metastases in patients with colorectal cancer [1]. In the past, only 10% of patients with CRLM were eligible to surgery. New chemotherapy schemes and improvement in surgical techniques allow to treat surgically patients with CRLM in advanced stages of illness [1]. Radioembolization (RE) using yttrium-90 (^90^Y) resin or glass microspheres, also known as selective internal radiation therapy (SIRT), is a palliative treatment [2] which reduces the liver tumour mass, eventually permitting surgical resection.

Clinical evaluation of patients with CRLM needs many different diagnostic tools. Morphological imaging procedures like computed tomography (CT), contrast enhanced CT (CECT), and magnetic resonance imaging (MRI) are useful techniques in staging and treatment evaluation of patients with CRLM, referring to the Response Evaluation Criteria In Solid Tumours (RECIST) [3]. Angiography allows the evaluation of vascular anatomy of the liver before SIRT. Single-photon emission computed tomography (SPECT) with Technetium ^99m^Tc albumin aggregated (^99m^Tc-MAA) is used to calculate hepatic shunts to other organs (as lungs) before or after SIRT [4].

Fluorine-18-fluorodeoxyglucose positron emission tomography and positron emission tomography/computed tomography (FDG-PET and PET/CT) are noninvasive functional imaging techniques which have become well established tools in an oncology setting [5]. FDG is a glucose analogue that identifies areas of high-glucose metabolism. Recently published PET response criteria (PERCIST) assessed the usefulness of FDG-PET and PET/CT in treatment evaluation of cancer patients [6].

Until now, several studies have shown the potential role of whole-body FDG-PET or PET/CT in patients with CRLM undergoing SIRT [7–25]. Therefore, the aim of our evidence-based paper is to provide a first evidence-based review of the literature on this topic to confirm known evidence data and to eventually investigate new emerging roles of FDG-PET or PET/CT in patients with CRLM undergoing SIRT.

## 2. Methods

### 2.1. Search Strategy

A comprehensive computer literature search of the PubMed/MEDLINE, Scopus, and Embase databases was conducted to find relevant published articles on whole-body FDG-PET or PET/CT in patients with CRLM undergoing SIRT with ^90^Y microspheres.

We used a search algorithm that was based on a combination of the terms (a) “SIRT” or “radioembolization” or “yttrium” and (b) “positron emission tomography” or “PET.” No beginning date limit was used; the search was updated until June 18, 2013. To expand our search, references of the retrieved articles were also screened for additional studies.

### 2.2. Study Selection

Studies or subsets in studies investigating the role of whole-body FDG-PET or PET/CT in patients with CRLM undergoing SIRT with ^90^Y were eligible for inclusion. Review articles, editorials or letters, comments, conference proceedings, case reports, and preclinical studies were excluded from this review.

Only those studies or subsets in studies that satisfied all of the following criteria were included: (a) FDG-PET and SIRT with ^90^Y performed in patients with CRLM, (b) sample size of at least ten patients with CRLM, and (c) only patients with histologically confirmed CRLM.

The exclusion criteria were (a) FDG-PET or SIRT with ^90^Y not performed in patients with CRLM, (b) sample size of less than ten patients with CRLM, and (c) studies with no histologically confirmed CRLM. The studies including patients with both liver metastases from colon-rectum cancer and different primary tumours (or with primary liver cancer) were excluded from this review, if data about CRLM could not be retrieved, to avoid bias in the literature data discussion.

Two researchers (S. Annunziata and G. Treglia) independently reviewed titles and abstracts of the retrieved articles, applying the inclusion and exclusion criteria mentioned above. Articles were rejected if they were clearly ineligible. The same two researchers then independently reviewed the full-text versions of the remaining articles to determine their eligibility for inclusion. Disagreements were resolved in a consensus meeting.

### 2.3. Data Abstraction

For each included study, information was collected concerning basic study (authors, journal, year of publication, country of origin, and type of study), patient characteristics (number of patients with MLT treated with SIRT, sex, mean age, and number of patients performing PET), and technical aspects (PET device, PET tracers, injected FDG activity, acquisition time, type of image analysis, ^90^Y device, and injected ^90^Y activity). Finally, the main findings of the articles included in this review have been reported and discussed.

## 3. Results

The comprehensive computer literature search from the PubMed/MEDLINE, Embase, and Scopus databases revealed 268 articles.

Nineteen articles comprising a total sample size of 833 patients with liver metastases were selected applying the inclusion criteria mentioned above [7–25]. Twenty studies involved patients with CRLM and patients with metastases from different primary tumours (or with primary liver cancer); thus they were excluded from this review [26–45].

**Figure 1:**
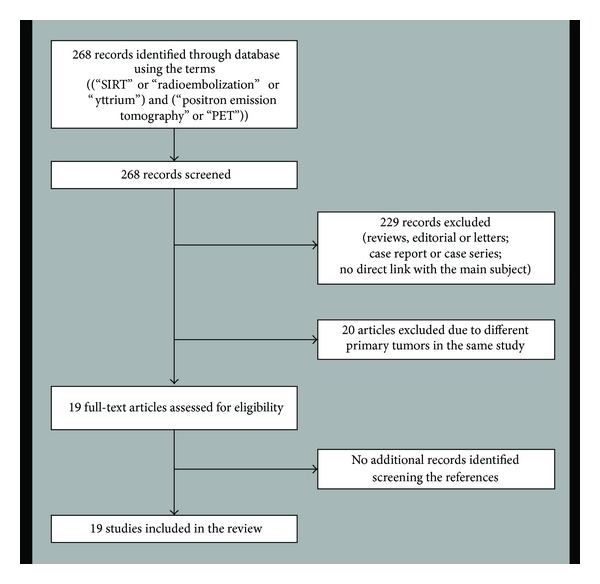
Flow chart of the search for eligible studies on the role of ^18^F-FDG-PET or PET/CT in patients with CRLM undergoing SIRT.

The 19 included studies were retrieved in their full-text version and included in this review. No additional studies were found after screening the references (Figure 1). The basic and technical characteristics of the studies included are shown in Tables 1 and 2.

### 3.1. Role of FDG-PET and PET/CT in Treatment Planning of Patients with CRLM Undergoing SIRT

FDG-PET or PET/CT may be used to stage patients with CRLM [46]. Until now, the role of these diagnostic tools in treatment planning of patients with CRLM undergoing SIRT is controversial [11, 12, 19, 23].

About the use of FDG-PET or PET/CT before SIRT, Denecke et al. [11] evaluated a standardized diagnostic approach using different radiological and nuclear medicine imaging procedures. All patients initially underwent chest and abdominal CT. Patients in whom CT revealed no contraindications against RE entered the next diagnostic step, which consisted of MRI of the liver and FDG-PET or PET-CT. This sequential diagnostic algorithm allowed an appropriate patient selection for RE of CRLM and reduced the number of unnecessary examinations and treatments. A recent study [23] included a total of 42 patients planned for SIRT. Patients who underwent both CT and FDG-PET in the diagnostic workup were selected. Findings on CT and FDG-PET matched in 20 patients. In 4 patients, lesions detected on CT were not FDG-avid, and in 18 patients FDG-PET showed significantly more lesions than CT. The same study [23] assessed the value of FDG-PET for preprocedural workup of patients with CRLM referred for RE and found that the use of FDG-PET changed patients' management in 7 out of 42 patients (17%). Six patients were not treated with RE because of extensive extrahepatic lesions that were only detected with FDG-PET. In one patient, abdominal CT had shown only one liver lesion that would have been treated with SIRT if it had not been for FDG-PET imaging which showed a second lesion in another segment.

In two recent studies FDG-PET or PET/CT was firstly used to validate other triage methods of patients planned for SIRT (as different formula of absorbed dose [12] or ^90^Y-PET imaging [19]). An American study group [12] evaluated a patient-specific SPECT-based method of dose calculation for treatment planning of SIRT. Absorbed dose to tumour and normal liver tissue was calculated by partition methods with two different tumour/normal liver vascularity ratios: an average 3 : 1 and a patient-specific ratio derived from pretreatment ^99m^Tc-MAA SPECT. Tumour dose calculated with the patient-specific method was more predictive of response ^90^Y SIRT. Differently, Bagni et al. [19] demonstrated the feasibility of ^90^Y-PET imaging for biodistribution assessment after SIRT. CRLM detected with ^90^Y-PET and ^99m^Tc-MAA were assessed and compared with FDG-PET obtained before treatment. ^90^Y-PET images were more accurate than ^99m^Tc-MAA SPECT, which is now considered the gold standard reference for biodistribution assessment.

Finally, FDG-PET or PET/CT before SIRT in patients with CRLM may have a role in staging of these patients and as baseline scan to further evaluate response to treatment. Further studies are needed to assess the role of FDG-PET or PET/CT in treatment planning and patients' selection before SIRT in patients with CRLM.

### 3.2. Role of FDG-PET and PET/CT in Treatment Evaluation of Patients with CRLM Treated with SIRT

Six studies included in this review analysed the role of FDG-PET and PET/CT in treatment evaluation of patients with CRLM treated with SIRT [7–10, 13, 14].

Firstly, FDG-PET or PET/CT was included in a diagnostic algorithm to evaluate feasibility, safety, and tumour response of patients with CRLM undergoing SIRT. These outcomes were investigated in glass microspheres labelled with ^90^Y [7, 8] and in different countries (as USA [9, 14] and Europe [10, 13]).

Recently, six studies mainly analysed the diagnostic performance of FDG-PET or PET/CT in treatment evaluation of patients with CRLM treated with SIRT [7–9, 13, 14, 22]. Several studies have shown that FDG-PET/CT imaging in liver metastases from different primary tumours performs better than anatomical imaging in evaluating the early tumour response to SIRT [25]. Wong et al. [7] assessed the feasibility of using FDG-PET for quantifying metabolic response of SIRT for CRLM by comparing visual estimates with hepatic lesions standardized uptake values (SUVs). SUVs of the entire axial slices of liver agree well with subjective visual evaluations, so quantitative FDG-PET is a useful technique in the treatment response evaluation of these patients. Lewandowski et al. [8] treated 27 patients with unresectable CRLM at a targeted absorbed dose of 135–150 Gy. Tumor response measured by FDG-PET imaging exceeded that measured by CT imaging for the first (88% versus 35%) and second (73% versus 36%) treated lobes. Another group [14] performed a baseline CT scan within 4 weeks of treatment. Baseline FDG-PET imaging was encouraged but not mandatory. Improvement was noted in 30 of 39 patients (77%) who had pretreatment and posttreatment FDG-PET studies available. Kennedy et al. [9] performed salvage SIRT for patients with unresectable CRLM that were refractory to oxaliplatin and irinotecan. A total of 208 patients were treated from April 2002 to April 2005. CT partial response was 35% and FDG-PET response was 91%. An Italian group [13] evaluated the effectiveness of CRLM RE with ^90^Y. A CT scan and a FDG-PET were performed to assess liver disease and to evaluate extrahepatic metastatic disease. According to RECIST, a complete response was observed in 2 patients, a partial response in 17 patients, stable disease in 14 patients, and progressive disease in 8 patients. Zerizer et al. [22] evaluated the ability of FDG-PET/CT imaging to predict early response to ^90^Y-RE in comparison with CECT using RECIST and lesion density criteria. The patients response to treatment were categorized using PET criteria, tumour density criteria, and RECIST. Early response assessment to ^90^Y-RE using FDG-PET/CT was superior to RECIST and tumour density.

In two different studies [16, 21], Tochetto et al. concluded that changes in CT attenuation of CRLM treated with ^90^Y-RE correlated highly with metabolic activity at FDG-PET and might be useful as an early surrogate marker for assessing treatment response. In the first study [16], for an attenuation reduction level of 15% or greater, attenuation showed 84% sensitivity and 83% specificity in predicting response at FDG-PET evaluation. In the second study [21], a similar strong association was found between FDG-PET response at 3 months and response based on attenuation criteria.

In another study [18], FDG-PET/CT was used to analyse patients treated with SIRT, in combination with contemporary systemic chemotherapy. Systemic chemotherapy was supplied to both liver lobes, whereas SIRT was administered selectively to the target liver lobe only. Response to treatment was evaluated by serial FDG-PET/CT performed at 4 weeks, 2 to 4 months, and 6 to 8 months. The chemo-SIRT combination produced greater objective responses as compared with chemo-only therapy in a front-line treatment in patients with CRLM.

Finally, in the past FDG-PET and PET/CT helped to assess that SIRT is a safe and feasible treatment for patients with CRLM. Recent studies confirmed that FDG-PET and PET/CT are useful to evaluate treatment response in these patients, in both early and long-term follow-up.

### 3.3. Prognostic Value of FDG-PET/CT in Patients with CRLM Undergoing SIRT

Five recent studies mainly focused on the prognostic value of FDG-PET/CT in patients with CRLM treated with SIRT [15, 17, 20, 22, 24].

In 2011 Gulec et al. [17] investigated the relationship between functional tumour volume (FTV), total lesion glycolysis (TLG), and clinical outcomes. FTV and TLG seemed to be predictive of clinical outcomes and useful criteria for patient selection and disease prognostication. In a recent study, authors of [25] evaluated tumour response using FDG-PET/CT in similar patients. Calculation of SUV, FTV, and TLG before and at the sixth week after SIRT seemed to play an important role in evaluating early tumour response and survival expectancy in these patients and to decide whether these patients should be referred to other treatment modalities or to follow-up. Another study [15] analysed 48 patients with pretreatment FDG-PET or PET/CT to find a score named tumour metabolic load index (TMLI), obtained converting SUV by logarithm in equivalent volumes of liver mass. TMLI value seemed to correlate with an increased occurrence of extrahepatic disease in patients with CRLM undergoing SIRT. Fendler et al. [24] confirmed this conclusion, assessing that changes in TLG rate predicted survival in patients with CRLM, whereas changes in SUV and RECIST criteria did not. Similarly, Zerizer et al. [22] evaluated the role of early FDG-PET/CT in predicting liver progression-free survival. Early FDG-PET/CT seemed to be superior to CECT in predicting progression-free survival in patients with liver metastases and tumour marker responses after ^90^Y-RE. To assess by FDG-PET/CT patterns of failure and factors affecting recurrence patterns, another group [20] demonstrated that patients with CRLM treated with SIRT developed a greater proportion of extrahepatic failure, and tumour volumes >300 mL were predictive for hepatic recurrence.

To date, FDG-PET/CT is an emerging prognostic tool in patients with CRLM undergoing SIRT. Semiquantitative factors (as FTV and TLG) seem to correlate with outcome and survival in these patients better than RECIST criteria. However, further prospective studies are needed to confirm this indication of FDG-PET/CT in patients with CRLM treated with SIRT.

### 3.4. Limitations of the Studies Included

Some limitations of the included studies in our evidence-based review should be underlined. The most significant ones are the heterogeneity of the patients enrolled, the variability in the sample analysed, the different devices used (PET or PET/CT, resin or glass ^90^Y-labelled microspheres), the methodology used to perform the scans (and lack of technical data in some papers), and the frequent retrospective nature of the studies. The most important limitation of this review is the exclusion of the studies including patients with both liver metastases from colon-rectum cancer and different primary tumours, to avoid possible biases in the literature data discussion.

## 4. Conclusion

From this first evidence-based review of the literature about the role of FDG-PET and PET/CT in patients with CRLM undergoing SIRT we conclude the following.

1. FDG-PET and PET/CT seem to be useful molecular imaging methods in evaluating treatment response of patients with CRLM treated with SIRT.
2. FDG-PET or PET/CT may have a role in treatment planning and patient selection for SIRT, but more studies are needed to confirm this indication.
3. FDG-PET/CT is emerging as an important prognostic tool in patients with CRLM undergoing SIRT, especially referring to PET semiquantitative analysis factors (as FTV and TLG).

Further studies are needed to evaluate the impact of FDG-PET and PET/CT on clinical management of these patients.

## Figures and Tables

**Table 1:** Basic characteristics of the included papers.

Authors	Year	Country	Type of study	Number of patients planned for SIRT	Sex (% male)	Mean age (years)	Number of patients undergoing PET
Wong et al. [7]	2004	USA	Prospective	27	56%	68	27
Lewandowski et al. [8]	2005	USA	NR	27	56%	68	27
Kennedy et al. [9]	2006	USA	Retrospective	208	62%	62	NR
Mancini et al. [10]	2006	Italy	Prospective	48	NR	Range 18–75	NR
Denecke et al. [11]	2008	Germany	NR	22	68%	58	18
Campbell et al. [12]	2009	USA	Retrospective	12	58%	Range 40–69	12
Cianni et al. [13]	2009	Italy	Retrospective	41	73%	61	NR
Mulcahy et al. [14]	2009	USA	Prospective	72	65%	61	39
Wong et al. [15]	2010	USA	Retrospective	48	58%	62	48
Tochetto et al. [16]	2010	USA	Retrospective	28	64%	62	28
Gulec et al. [17]	2011	USA	Prospective	20	65%	61	20
Gulec et al. [18]	2013	USA	Prospective	20	65%	61	20
Bagni et al. [19]	2012	Italy	NR	10	60%	63	10
Schonewolf et al. [20]	2012	USA	Retrospective	30	60%	61	30
Tochetto et al. [21]	2012	USA	Retrospective	38	65%	65	20
Zerizer et al. [22]	2012	UK	Retrospective	25	56%	59	25
Rosenbaum et al. [23]	2013	Netherlands	Retrospective	42	57%	59	42
Fendler et al. [24]	2013	Germany	Prospective	80	73%	61	80
Soydal et al. [25]	2013	Turkey	NR	35	57%	62	NR

NR: not reported.

**Table 2:** Technical characteristics of the included papers.

Authors	Year	PET device	FDG mean injected activity (MBq)	Time between injection and acquisition (min)	PET image analysis	^ 90^Y-SIRT device	^ 90^Y mean injected activity (GBq)
Wong et al. [7]	2004	PET	370	60	Visual and semiquantitative	Glass microspheres	LM formula
Lewandowski et al. [8]	2005	PET	370	60	Visual	Glass microspheres	2.37
Kennedy et al. [9]	2006	PET	NR	NR	Visual	Resin microspheres	1.75
Mancini et al. [10]	2006	PET	NR	NR	Visual	Resin microspheres	BSA method
Denecke et al. [11]	2008	PET or PET/CT	4-5/kg	90	Visual	NR	NR
Campbell et al. [12]	2009	PET/CT	555	Range 60–90	Visual and semiquantitative	Resin microspheres	0.92
Cianni et al. [13]	2009	PET	NR	NR	Visual	Resin microspheres	1.82
Mulcahy et al. [14]	2009	PET	NR	NR	Visual	Glass microspheres	NR
Wong et al. [15]	2010	PET or PET/CT	Range 370–555	60	Visual and semiquantitative	NR	NR
Tochetto et al. [16]	2010	PET	360	60	Visual and semiquantitative	Glass microspheres	NR
Gulec et al. [17]	2011	PET/CT	NR	NR	Visual and semiquantitative	Resin microspheres	1.58
Gulec et al. [18]	2013	PET/CT	NR	NR	Visual and semiquantitative	Resin microspheres	1.58
Bagni et al. [19]	2012	PET/CT	NR	NR	Visual and semiquantitative	Resin microspheres	1.37
Schonewolf et al. [20]	2012	PET/CT	NR	NR	Visual and semiquantitative	Resin microspheres	1.85
Tochetto et al. [21]	2012	PET	360	60	Visual and semiquantitative	Glass microspheres	NR
Zerizer et al. [22]	2012	PET/CT	370	60	Visual and semiquantitative	Resin microspheres	BSA method
Rosenbaum et al. [23]	2013	PET or PET/CT	3.7/kg	60	Visual	NR	NR
Fendler et al. [24]	2013	PET/CT	300	60	Visual and semiquantitative	Resin microspheres	1.80
Soydal et al. [25]	2013	PET/CT	Range 296–370	60	Visual and semiquantitative	Resin microspheres	BSA method

NR: not reported, BSA: body surface area, and LM: liver mass.
